# Delayed Relapse of Gastric Gastrointestinal Stromal Tumor After 17 Years: A Case Report and Review of Surveillance Challenges

**DOI:** 10.7759/cureus.92783

**Published:** 2025-09-20

**Authors:** Irfan Ullah, Hafiz A Haseeb, Rachel Puw-Jones, Saad Arif, Mohanad Alhasan, Giustino Varrassi

**Affiliations:** 1 Gastroenterology and Hepatology, University Hospital of Wales, Cardiff, GBR; 2 Gastroenterology, Grange University Hospital, Cwmbran, GBR; 3 Pathology, Aneurin Bevan University Health Board, Newport, GBR; 4 Gastroenterology and Hepatology, Glangwili Hospital, Carmarthen, GBR; 5 Gastroenterology and Hepatology, Prince Philip hospital, Swansea, GBR; 6 Pain Medicine, Fondazione Paolo Procacci, Rome, ITA

**Keywords:** cd117, gastrointestinal stromal tumor (gist), multidisciplinary approach (mdt), platelet-derived growth factor receptor alpha (pdgfra) mutation, rare cancers

## Abstract

Gastrointestinal stromal tumors (GISTs) are rare mesenchymal neoplasms. Complete surgical resection is the mainstay of treatment, yet recurrence remains common even after apparent complete resection. Gastrointestinal stromal tumors carry a notable long-term risk of recurrence even after surgery, underscoring the need for ongoing vigilance. We present a case of very late recurrence of gastric GIST 17 years after complete resection. This case highlights the limitations of current surveillance protocols and raises important questions about tumor biology, genetic risk stratification, and the need for long-term or even lifelong follow-up in selected patients.

## Introduction

Gastrointestinal stromal tumors (GISTs) represent the most common mesenchymal tumors of the gastrointestinal tract, comprising approximately 3% to 5% of all soft tissue sarcomas and 1% to 2% of gastrointestinal cancers [1]. They arise most frequently in the stomach (50% to 70%) and small intestine (20% to 30%), with fewer cases in the colon, rectum, and esophagus. Clinically, they present with gastrointestinal bleeding, abdominal pain, or an incidental mass, most commonly in the stomach or small intestine [2]. Most GISTs exhibit activating mutations in v-kit Hardy-Zuckerman 4 feline sarcoma viral oncogene homolog (KIT) or platelet-derived growth factor receptor alpha (PDGFRA), with CD117, also known as c-KIT, and discovered on GIST-1 (DOG1), serving as reliable diagnostic markers [3].

Complete surgical resection remains the cornerstone of treatment for localized GIST. Adjuvant imatinib therapy has become standard for high-risk patients, improving recurrence-free survival, as it specifically targets the CD117 and PDGFRA mutations that drive tumor growth in GIST [4]. Despite these advances, recurrence remains common, with reported 10-year recurrence-free survival ranging between 54% and 60% [5]. Risk stratification based on tumor size, site, and mitotic index informs prognosis, but there is no universally accepted guideline for long-term surveillance imaging [6]. Delayed recurrence, defined as recurrence more than 10 years after resection, is rare but clinically significant. Such cases highlight gaps in current follow-up strategies and raise questions about tumor biology, genetic heterogeneity, and the adequacy of existing risk models [7].

## Case presentation

A 54-year-old woman was referred by her general practitioner with symptoms of heartburn and suspected incisional hernia at the site of prior gastric surgery for GIST in 2007. She reported a one-year history of progressive abdominal distension associated with mild discomfort. Her past medical history included hypertension and a hysterectomy for uterine fibroids.

In 2007, the patient was incidentally found to have a large gastric mass during evaluation for menorrhagia. A CT revealed a 13 × 16 × 12 cm lesion arising from the posterior gastric wall and greater curvature, abutting the pancreas and transverse colon. She was asymptomatic at the time, with an Eastern Cooperative Oncology Group (ECOG) performance status of 0. She underwent complete surgical resection with an uncomplicated recovery.

Histopathology confirmed GIST with central necrosis, low mitotic activity (<5/50 high-power fields), and clear resection margins. Adjuvant imatinib was not initiated, as per the multidisciplinary team (MDT) decision, given the low mitotic rate. Surveillance CT scans were performed for two years, which were unremarkable, after which the patient was lost to follow-up from 2010 to 2025.

**Figure 1:**
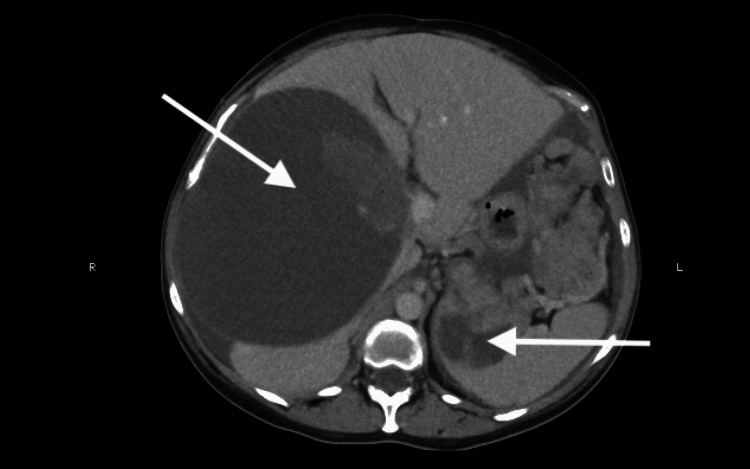
CT hepatic and splenic cystic metastasis with multiple intraperitoneal deposits Contrast-enhanced CT of the abdomen showing widespread peritoneal deposits, cystic hepatic and splenic metastases, and distortion of the pancreatic outline, consistent with recurrent GIST. GIST: Gastrointestinal stromal tumor

At her current presentation in 2025, the abdominal examination revealed moderate ascites. The CT imaging demonstrated widespread peritoneal deposits of varying size, cystic hepatic and splenic metastases, and distortion of the pancreas (Figure 1).

**Figure 2:**
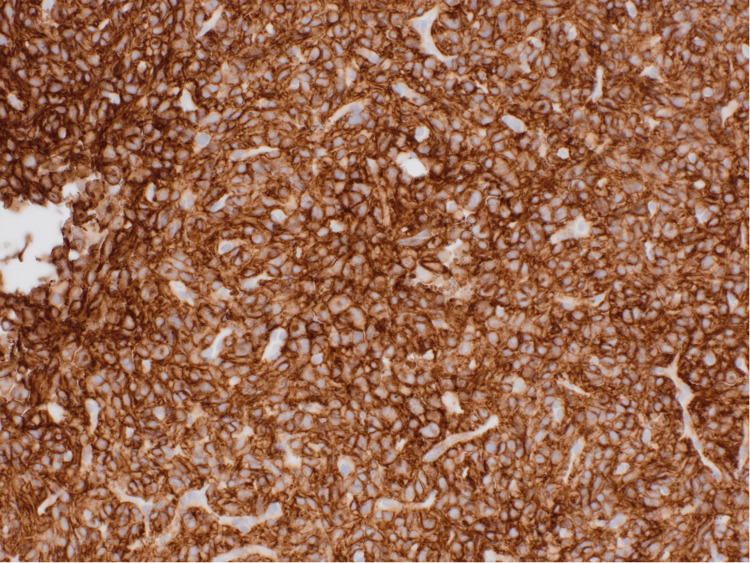
Immunohistochemistry demonstrating diffuse membranous and cytoplasmic staining for CD117 at 20x magnification The CD117 is a sensitive marker for GIST, expressed in up to 95% of cases. The CD117-negative tumors are most often gastric in origin, frequently show epithelioid morphology, and typically harbor PDGFRA mutations. GIST: Gastrointestinal stromal tumors, PDGFRA: Platelet-derived growth factor receptor alpha

**Figure 3:**
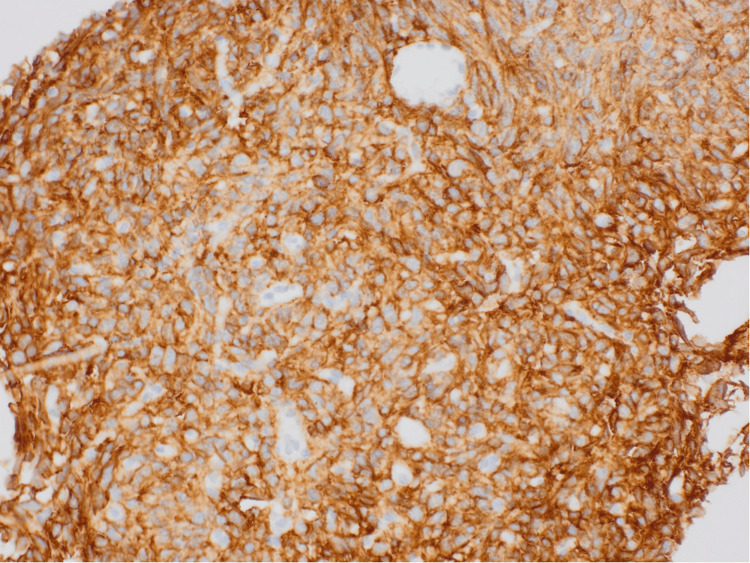
Immunohistochemistry showing diffuse, strong membranous and cytoplasmic staining for DOG1 at 20x magnification The DOG1 is a highly sensitive and specific marker for GIST, with reported positivity in up to 97% of cases. Positive expression helps distinguish GIST from intra-abdominal (desmoid) fibromatosis, nerve sheath tumors, and melanoma. DOG1: Discovered on GIST-1, GIST: Gastrointestinal stromal tumors

Ultrasound-guided biopsy of the peritoneal lesions showed spindle and epithelioid cells with positive immunohistochemical staining for CD117/c-KIT proto-oncogene receptor tyrosine kinase; CD34; DOG1 (also known as anoctamin-1); B-cell lymphoma protein (BCL, typically BCL-2); c-KIT, synonymous with CD117; and recepteur d’origine nantais-1 (RON-1)/macrophage stimulating 1 receptor (MST1R) tyrosine kinase (Figures 2-3). The tumor cells were negative for smooth muscle actin (SMA), desmuslin (desmin, a muscle-specific intermediate filament protein), and cytokeratin AE1/AE3 antibody cocktail (AE1/3 is a broad-spectrum keratin marker). There was a loss of succinate dehydrogenase complex, subunit B (SDHB) expression. No mitoses were identified in the biopsy sample. Molecular testing was negative for v-Raf murine sarcoma viral oncogene homolog B1 valine to glutamic acid substitution at codon 600 (BRAF V600E mutation) and neurotrophic tyrosine receptor kinase (NTRK) translocation.

**Figure 4:**
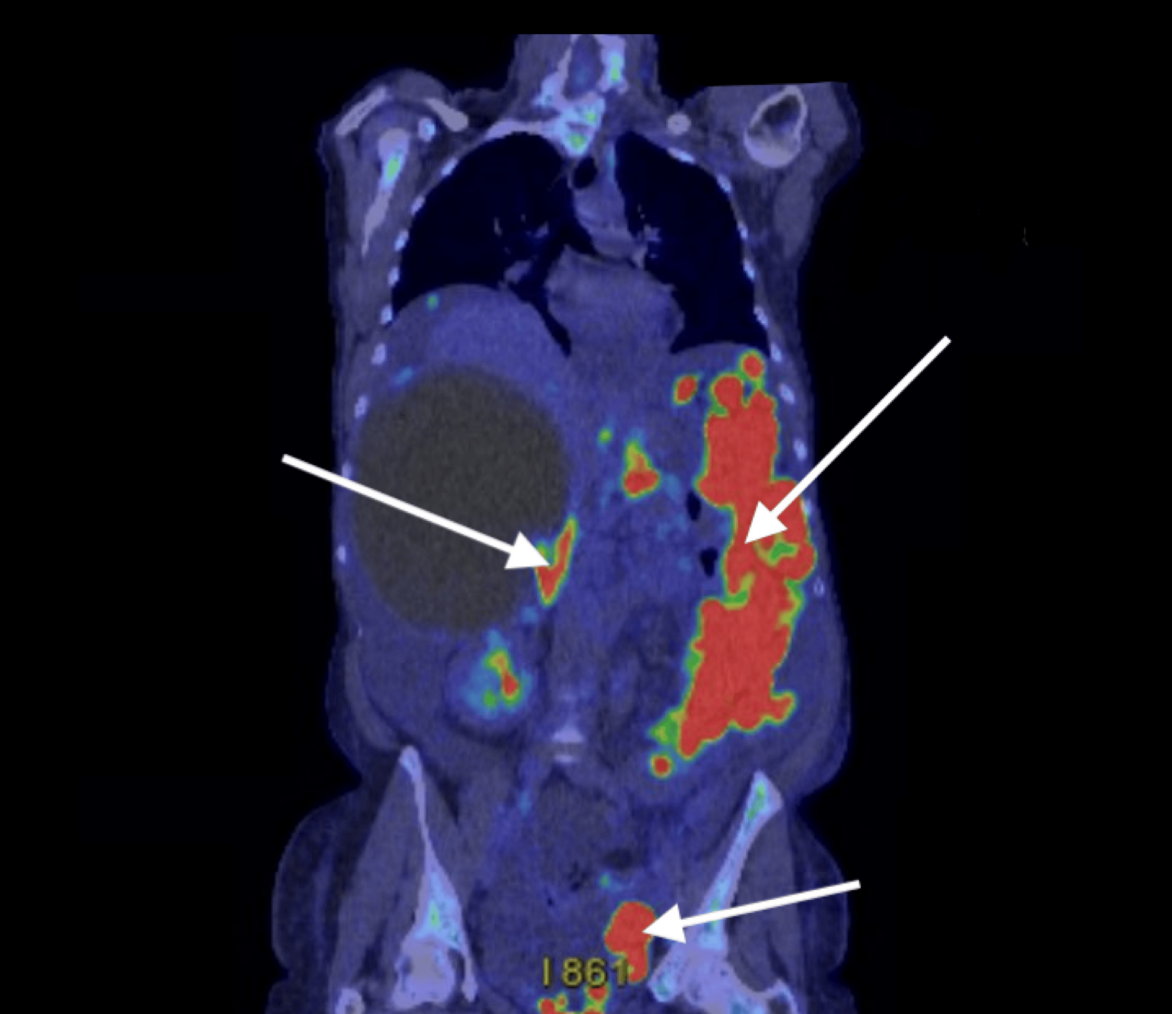
PET-CT showing extensive peritoneal infiltration with heterogeneous FDG uptake Cystic hepatic and splenic metastases demonstrate variable tracer activity. No FDG-avid disease was observed above the diaphragm. FDG: Fluorodeoxyglucose

As demonstrated on PET-CT (Figure 4), the patient had widespread peritoneal infiltration with mixed fluorodeoxyglucose (FDG) uptake, along with cystic hepatic and splenic metastases showing heterogeneous tracer activity. No FDG-avid disease was identified above the diaphragm.

Based on the patient’s history, imaging, and immunohistochemistry, a diagnosis of recurrent metastatic GIST (wild type) was made. The patient was referred to the oncology service. She was commenced on imatinib 400 mg once daily with palliative intent, given the extent of peritoneal disease and the long disease-free interval. Ascites was managed conservatively to assess therapeutic response, as drainage was not feasible due to the extensive peritoneal involvement. At the one-month follow-up, the patient remained clinically stable and tolerated imatinib without significant adverse effects. At the time of reporting this case, a repeat PET-CT was scheduled for the third month to assess treatment response.

## Discussion

Most GIST recurrences occur within the first five years after resection, but delayed relapse remains a recognized, though rare, phenomenon. The present case, with recurrence 17 years after complete resection of a gastric GIST, underscores the difficulty in predicting long-term tumor behavior and highlights the limitations of current surveillance strategies [8].

The GIST biology is strongly influenced by its underlying genetic profile. The majority of tumors are driven by KIT or PDGFRA mutations, and certain genotypes, such as KIT exon 11 mutations, have been linked to a higher recurrence risk even after complete resection [8]. In contrast, KIT/PDGFRA wild-type tumors, which account for 10% to 15% of cases, often demonstrate unique clinical behavior and unpredictable courses. Loss of SDH expression, as observed in this patient, is increasingly recognized as a feature of a distinct subgroup with variable outcomes and potential for late relapse [6-7].

Tumor dormancy may also contribute to very late recurrence. Microscopic residual disease can persist undetected for years, influenced by immune surveillance, angiogenesis, and the tumor microenvironment. Reactivation of these dormant cells may explain recurrences long after initial treatment. In this patient, the absence of adjuvant therapy likely compounded this risk. At the time of her initial diagnosis in 2007, adjuvant imatinib was not widely adopted for intermediate-risk tumors. Today, however, guidelines recommend at least three years of adjuvant therapy for high-risk disease, which has been shown to significantly reduce recurrence rates [4]. In retrospect, the patient's large primary tumor (>10 cm) would likely have justified more aggressive adjuvant management, regardless of the low mitotic index.

The implications for clinical practice are significant. Current surveillance recommendations advise imaging every three to six months for the first three years and annually thereafter [2,6]. However, the optimal duration of follow-up remains undefined. This case illustrates that even patients with low mitotic index tumors can experience recurrence after very long disease-free intervals, suggesting that lifelong surveillance should be considered in selected patients, particularly those with large gastric primaries.

Finally, advances in molecular profiling may help refine risk prediction and surveillance strategies. Identifying subgroups such as SDH-deficient and KIT/PDGFRA-negative tumors emphasizes the need for more individualized management. In addition, ongoing research into secondary mutations and novel therapeutic targets may expand treatment options for patients with late or resistant disease [7,8].

This case of a 17-year delayed recurrence of gastric GIST highlights the importance of integrating clinical, histologic, and molecular risk factors into long-term follow-up planning. It also underscores the urgent need for consensus guidelines on extended surveillance and tailored use of adjuvant therapy.

## Conclusions

Delayed recurrence of GIST, even decades after resection, remains a clinical reality. This case highlights the need for lifelong surveillance in selected patients, particularly those with large primary tumors, regardless of initial low mitotic index. Long-term studies are needed to refine surveillance protocols, incorporate genetic risk factors, and optimize the role of adjuvant therapy.
